# Increased light‐enhanced dark respiration under warming suggests intensified metabolic coupling in an Arctic diatom

**DOI:** 10.1111/nph.70359

**Published:** 2025-07-04

**Authors:** Linda Rehder, Björn Rost, Sven A. Kranz, Sebastian D. Rokitta

**Affiliations:** ^1^ Marine Biogeosciences Alfred‐Wegener‐Institute ‐ Helmholtz Centre for Polar and Marine Research 27570 Bremerhaven Germany; ^2^ Faculty of Biology/Chemistry University of Bremen 28359 Bremen Germany; ^3^ Department of BioSciences Rice University 77005 Houston TX USA

**Keywords:** alternative electron flow, photosynthesis, phytoplankton, plastidial–mitochondrial coupling, primary production, respiration, temperature responses

## Disclaimer

The New Phytologist Foundation remains neutral with regard to jurisdictional claims in maps and in any institutional affiliations.

## Introduction

Temperature affects functional traits such as growth rates, biomass production or cellular quotas in Arctic phytoplankton to different degrees, likely resulting from diverging temperature sensitivities of underlying physiological key processes. We used membrane‐inlet mass spectrometry to measure ^16^O_2_, ^18^O_2_ and carbon dioxide (CO_2_) fluxes to quantify photosynthetic O_2_ production (${\mathrm{PS}}_{O_{2}}$), photosynthetic C‐fixation (${\mathrm{PS}}_{{\mathrm{CO}}_{2}}$), respiratory CO_2_ release ($R_{{\mathrm{CO}}_{2}}$), respiratory O_2_ consumption in the dark ($R_{O_{2}}$) and light‐enhanced dark respiration (LEDR) as well as fast repetition rate fluorometry to quantify quantum yields and electron transport in the Arctic diatom *Thalassiosira hyalina* under different warming scenarios. At higher temperatures, cells upregulate light harvesting abilities to compensate for a lower maximum photosystem II (PSII) quantum yield, thereby maintaining O_2_ production and likely also thylakoid proton motive force (PMF). Furthermore, substantial LEDR under warming suggests that plastidial reductant is increasingly rerouted towards mitochondrial O_2_ consumption to support a sufficient ATP : NADPH ratio inside chloroplasts. In line with the indicated reductant import, the mitochondrial citric acid cycle is suppressed, minimizing respiratory CO_2_ loss despite stimulated LEDR. This suggests that the optimization of the plastidial ATP : NADPH ratio is critical for higher net biomass retention under warming. Our findings underline the necessity to interpret subprocesses of photosynthesis and respiration independently and explain the often‐observed enhanced biomass accumulation in a warmer Arctic Ocean.

The Arctic Ocean is most prone to climate change, with warming rates being higher than the global average, especially in the Fram Strait area (Carvalho & Wang, 2020; Constable *et al*., 2022; Rantanen *et al*., 2022). In addition to the general trend of rising mean sea surface temperatures, marine heatwaves have notably increased in frequency, duration and intensity (Hobday *et al*., 2016; Oliver *et al*., 2019). Hence, the Arctic is already exposed to exceptional degrees of ocean warming, and model simulations project further temperature increases, especially in the ever extending sea‐ice‐free regions. Since phytoplankton represent the base of the marine food web and contribute significantly to marine biogeochemical cycling (Falkowski *et al*., 1998; Field *et al*., 1998), their responses to warming have the potential to impact the entire Arctic ecosystem.

Temperature as a universal driver affects molecular movement and consequently all physical and (bio)chemical reactions (Brown *et al*., 2004; Pearle *et al*., 2010), including also those key processes involved in primary production. As single‐celled phototrophic organisms, phytoplankton perform oxygenic photosynthesis to convert light energy into (bio)chemical energy (Falkowski & Raven, 2013; Falciatore *et al*., 2022). First, the excitation energy harvested by pigment molecules is used to successively extract electrons from water, which are subsequently passed on to the photosynthetic electron transport chain (ETC) and ultimately lead to the generation of reductants, that is, electron carriers like NADPH. With the flow of electrons along the ETC, a PMF is established, which is later exploited for the generation of ATP. Both ATP and NADPH subsequently fuel the Calvin cycle, where the enzyme Ribulose‐1,5‐bisphosphate‐carboxylase/‐oxygenase (RubisCO) catalyzes the fixation of CO_2_ into organic biomass. The ATP : NADPH ratio created in the photosynthetic light reactions, however, is typically too low to satisfy the stoichiometric demands of the Calvin cycle (Allen *et al*., 2005; Lepetit *et al*., 2022), so that cells experience an overreduction of the chloroplasts. Therefore, alternative electron flows (AEFs) are necessary to either increase the relative amount of ATP or to divert excess NADPH (Allen *et al*., 2005; Curien *et al*., 2016). AEFs comprise cyclic electron flow, which is known to be prominent in polar diatoms (Goldman *et al*., 2015), oxygen (O_2_) consuming water‐to‐water cycles, such as the Mehler reaction and the plastid terminal oxidase (PTOX). The metabolic coupling of the chloroplasts with other cellular compartments represents another mechanism, in which excess reductant from the plastid is dissipated to, for example, the cytoplasm and the mitochondria. All these processes either support the PMF, and thus ATP production, or dissipate reductants in order to increase the ATP : NADPH ratio (e.g. Asada, 2000; Bailleul *et al*., 2015; Curien *et al*., 2016; Nawrocki *et al*., 2019; Lepetit *et al*., 2022). Regarding temperature sensitivity, light harvesting is typically less sensitive than the use of ATP and NADPH in metabolic processes (Baker *et al*., 1988; Raven & Geider, 1988). Consequently, any temperature‐dependent change in the biochemical consumption of ATP and NADPH will require physiological adjustments on multiple levels. Therefore, AEFs can function as regulatory valves to maintain metabolic homeostasis inside the chloroplast and across cellular compartments, especially under stressful environmental conditions, as induced by increasing light intensities or temperatures, for example.

Ecophysiological processes, including cell division, photosynthesis and respiration, are typically stimulated by elevated temperature as a result of accelerated diffusion rates, membrane fluidity and enzyme activity (Los *et al*., 2013; Young *et al*., 2015; Padfield *et al*., 2016; Schuback *et al*., 2017). However, temperature‐induced changes are process‐specific (e.g. Baker *et al*., 2016; Barton *et al*., 2020; Rehder *et al*., 2024), and cells require physiological regulation, such as the above‐described AEFs, to overcome metabolic imbalances and to avoid detrimental temperature effects (Rehder *et al*., 2023). Consequently, continuous adjustment of physiological processes is critical for phytoplankton to maintain metabolic homeostasis over the widest possible range of experienced temperatures. Arctic phytoplankton has evolved to be surprisingly plastic toward ocean warming (Hoppe *et al*., 2018; Rehder *et al*., 2024; Wolf *et al*., 2024). Previous studies, for instance, found that many prominent Arctic phytoplankton species currently live substantially below their optimal temperatures and thus, it is expected that these organisms will benefit from moderate warming.

In this study, we hypothesized that Arctic phytoplankton modulate photophysiology as well as AEF operation to adjust photosynthetic and respiratory processes to warmer temperatures. To resolve these regulatory efforts, we acclimated the Arctic diatom *T. hyalina* to different temperatures (2°C, 6°C and 10°C) and measured photophysiological parameters as well as rates of ^16^O_2_ evolution, ^18^O_2_ uptake and CO_2_ fluxes by means of fast repetition rate fluorometry (FRRf) and membrane‐inlet mass spectrometry (MIMS), respectively.

## Materials and Methods

### Phytoplankton cultivation

We cultured the Arctic diatom *T. hyalina* (KB3 SS5, isolated 2021 in Svalbard, Norway) at 2°C, 6°C and 10°C in 0.2‐μm sterile‐filtered Arctic seawater (Salinity 31), enriched with vitamins and trace metals according to f/2 media (Guillard & Ryther, 1962). Nitrate, silicate and phosphate were added in concentrations of 100, 100 and 6 μmol l^−1^, respectively. The irradiance was set to continuous light at 30 μmol photons m^−2^ s^−1^ to mimic a polar day light climate. Cells were acclimated to experimental conditions for at least seven generations as semi‐continuous dilute batch cultures in aerated 2 l glass bottles (Schott Instruments, Mainz, Germany) under continuous supply of humidified air (pCO_2_ of 400 μatm) generated in a gas mixing system (CGM 2000; MCZ Umwelttechnik, Bad Nauheim, Germany). To ensure temperature stability, culture bottles were submersed in temperature‐controlled aquaria. Cell concentrations during cultivation never exceeded 8000 cells ml^−1^ to ensure nutrient replete conditions and stable carbonate chemistry.

### Growth, elemental composition and pigmentation

Specific growth rates were calculated from daily assessed cell concentrations obtained during the exponential growth phase, according to

(Eqn 1) $$\mu =\left(\left(\log_{e}\left(N_{1}\right)-\log_{e}\left(N_{0}\right)\right)/\left(t_{1}-t_{0}\right)\right)$$

where μ is the specific growth rate (d^−1^), and *N*_0_ as well as *N*_1_ are the cell concentrations at the initial and final time points *t*_0_ and *t*_1_, respectively. Counting was performed with a cell‐counter (Beckmann‐Coulter Multisizer III, Fullerton, USA).

Particulate organic carbon (POC) samples were filtered onto precombusted (12 h, 500°C) glass fiber filters (GF/F, 0.7 μm nominal pore size; Whatman, Maidstone, UK). After drying for at least 24 h at 60°C, filters were submitted to elemental analysis (EuroVector EA 3000, Pavia, Italy) using the flash combustion technique (Knap *et al*., 1996). Chl *a* samples were filtered onto precombusted (12 h, 500°C) GF/F (0.7 μm; Whatman), shock frozen in liquid nitrogen and stored at −80°C until extraction. Chl *a* was extracted overnight in 90% acetone (Sigma‐Aldrich) with an additional cell disruption using a cell‐mill (Precellys 24; Bertin, Montigny‐le‐Bretonneux, France). Extracts were centrifuged (13 000 ***g*** for 5 min; Sigma 4K10), and Chl *a* concentration in the supernatant was determined using the fluorometric ‘acidification method’ (Turner Trilogy Fluorometer; Turner Designs, San Jose, CA, USA; Knap *et al*. (1996)).

### Photophysiological parameters

Photophysiological characteristics of PSII were assessed by means of Chl *a* variable fluorescence using an FRRf (FastOcean; Chelsea Technologies, Yateley, UK) combined with the FastAct2 Laboratory system (Chelsea Technologies). Light‐emitting diodes were set to 450 nm emission wavelength to fully saturate all PSII reaction centers on short timescales. We used the FRRf in single turnover mode, in which the saturation phase comprised 100 flashlets on a 2 μs pitch, and the relaxation phase comprised 40 flashlets on a 60 μs pitch. All measurements were conducted in a temperature‐controlled cuvette at the respective acclimation temperature after dark acclimation for 45 min.

Minimum Chl *a* fluorescence (*F*_0_ and *F*_0_′ for dark‐ and light‐acclimated measurements, respectively) and maximum Chl *a* fluorescence (*F*_m_ and *F*_m_′ for dark‐ and light‐acclimated measurements, respectively) were obtained from iteratively fitting the induction phase (Kolber *et al*., 1998), and re‐opening times of PSII (τ) were obtained from iteratively fitting the relaxation phase (Oxborough, 2012). FRRf measurements were performed to obtain photosynthesis irradiance (PI) curves with eight light levels (5 min preacclimation at respective actinic light per light level; maximum light level was 720 μmol photons m^−2^ s^−1^). Basic photophysiological parameters, such as maximum quantum yields of PSII in the dark (*F*_v_/*F*_m_) and light ($F_{q}^{\prime }/F_{m}^{\prime }$; Supporting Information Table S1), the yields of regulated (Y(NPQ); Table S1) and nonregulated (Y(NO)) energy dissipation of PSII and the functional absorption cross sections of PSII in the dark‐regulated state (σ_PSII; dark_) were obtained using standard calculations (McKew *et al*., 2013; Schuback *et al*., 2017; Trimborn *et al*., 2017). Relative electron transport rates (rETRs) were calculated as

(Eqn 2) $$\text{rETR}=\frac{F_{q}^{\prime }}{F_{m}^{\prime }}\times I$$

Absolute electron transport rates (absETRs) were calculated as

(Eqn 3) $$\text{absETR}=\sigma_{\text{PSII};\text{dark}}\times \left(\frac{\frac{{F_{q}}^{\prime }}{F_{m}^{\prime }}}{\frac{F_{v}}{F_{m}}}\right)\times I$$

Light acclimation indices (*I*_k_) were calculated from absETRs following Trimborn *et al*. (2017). Optical antenna sizes (σ_LHCII_) were obtained by dividing σ_PSII; dark_ by (*F*_v_/*F*_m_).

### Gas flux measurements

A MIMS (Isoprime, GV Instruments, Manchester, UK) was used to measure (1) photosynthetic O_2_ production during light (${\mathrm{PS}}_{O_{2}}$), (2) respiratory O_2_ consumption during dark ($R_{O_{2}}$) and light (so‐called LEDR) and (3) photosynthetic net C‐fixation in the light (${\mathrm{PS}}_{{\mathrm{CO}}_{2}}$) and respiratory CO_2_ release in the dark ($R_{{\mathrm{CO}}_{2}}$). These rates were determined by measuring changes in gas concentrations of ^16^O_2_, ^18^O_2_ and CO_2_ within each biological replicate. Calibrations followed the procedures described in Rokitta & Rost (2012) for ^16^O_2_ and CO_2_. To calibrate for ^18^O_2_, a normal ^16^O_2_ calibration was performed and the acceleration voltage of the mass spectrometer was adjusted to redirect the ^16^O_2_ beam to the ^18^O_2_ detector. All fluxes were corrected for instrumental consumption of ^16^O_2_ and ^18^O_2_ under each temperature treatment. For ^18^O_2_ consumption measurements, the assay medium was purged with N_2_ gas overnight to remove all ^16^O_2_. Subsequently, the medium was transferred into the MIMS cuvette and equilibrated with ^18^O_2_ gas.

The bioassays were performed using concentrated cell suspensions. To this end, cells were concentrated by gentle filtration over polycarbonate filters (Isopore TSTP, 3 μm pore size, Merck, Darmstadt, Germany) and resuspended in O_2_‐ and CO_2_‐free culture medium buffered to a pH_NBS_ of 7.9 (50 mM HEPES). Subsequently, cells were transferred to the temperature‐ and light‐controlled MIMS cuvette and spiked with ^18^O_2_ reaching a final O_2_ concentration of *c*. 21%. Dissolved inorganic carbon (DIC) was added to the cuvette to yield typical concentration of seawater (*c*. 2200 μmol l^−1^). Carbonic anhydrase was added (500 μg l^−1^ final concentration) to ensure instantaneous equilibration of the carbonate system (Rokitta & Rost, 2012). Using the CO_2_ : DIC ratios obtained in the calibrations, CO_2_ traces were converted to total carbon fluxes. Flux measurements were performed in consecutive light–dark phases of 3 min each, at two light intensities (30 and 150 μmol photons m^−2^ s^−1^).

Calculations for net and gross rates of ${\mathrm{PS}}_{O_{2}}$, $R_{O_{2}}$ as well as LEDR ($R_{O_{2}}$ in the light) followed Fock & Sültemeyer (1989): Accordingly, we subtracted mean $R_{O_{2}}$ rates from the $R_{O_{2}}$ rates in the light to obtain LEDR per light level and temperature. Net C‐fixation rates were obtained from CO_2_ fluxes during the light phases, whereas rates of $R_{{\mathrm{CO}}_{2}}$ were obtained from CO_2_ fluxes during the dark phases. Gross rates of C‐fixation (${\mathrm{PS}}_{{\mathrm{CO}}_{2}}$) were calculated by subtracting mean $R_{{\mathrm{CO}}_{2}}$ rates from net C‐fixation rates during light. After the assays, duplicate Chl *a* samples were taken from the cuvette to express the obtained rates based on Chl *a*. Photosynthetic and respiratory quotients (PQ and RQ, respectively) were calculated from gross rates as PQ = ${\mathrm{PS}}_{O_{2}}$/${\mathrm{PS}}_{{\mathrm{CO}}_{2}}$ and RQ = $R_{{\mathrm{CO}}_{2}}$/$R_{O_{2}}$ according to Rehder *et al*. (2023).

### Statistical analysis

All acclimation and photophysiological parameters are presented as the mean of four biological replicates ± standard deviation, if not stated otherwise. Data obtained from gas flux measurements are presented in three biological replicates ± SD, except for the 2°C treatment (*n* = 2). For acclimation and photophysiological parameters, normal distribution was tested using the Shapiro–Wilk test, and equal variances were confirmed using the Welch test. We performed ANOVA followed by Tukey's *post hoc* test to test for significant differences using Jasp statistics (Love *et al*., 2019). The level of significance was set to *P* ≤ 0.05.

## Results

### Acclimation responses and photophysiological parameters

**Fig. 1:**
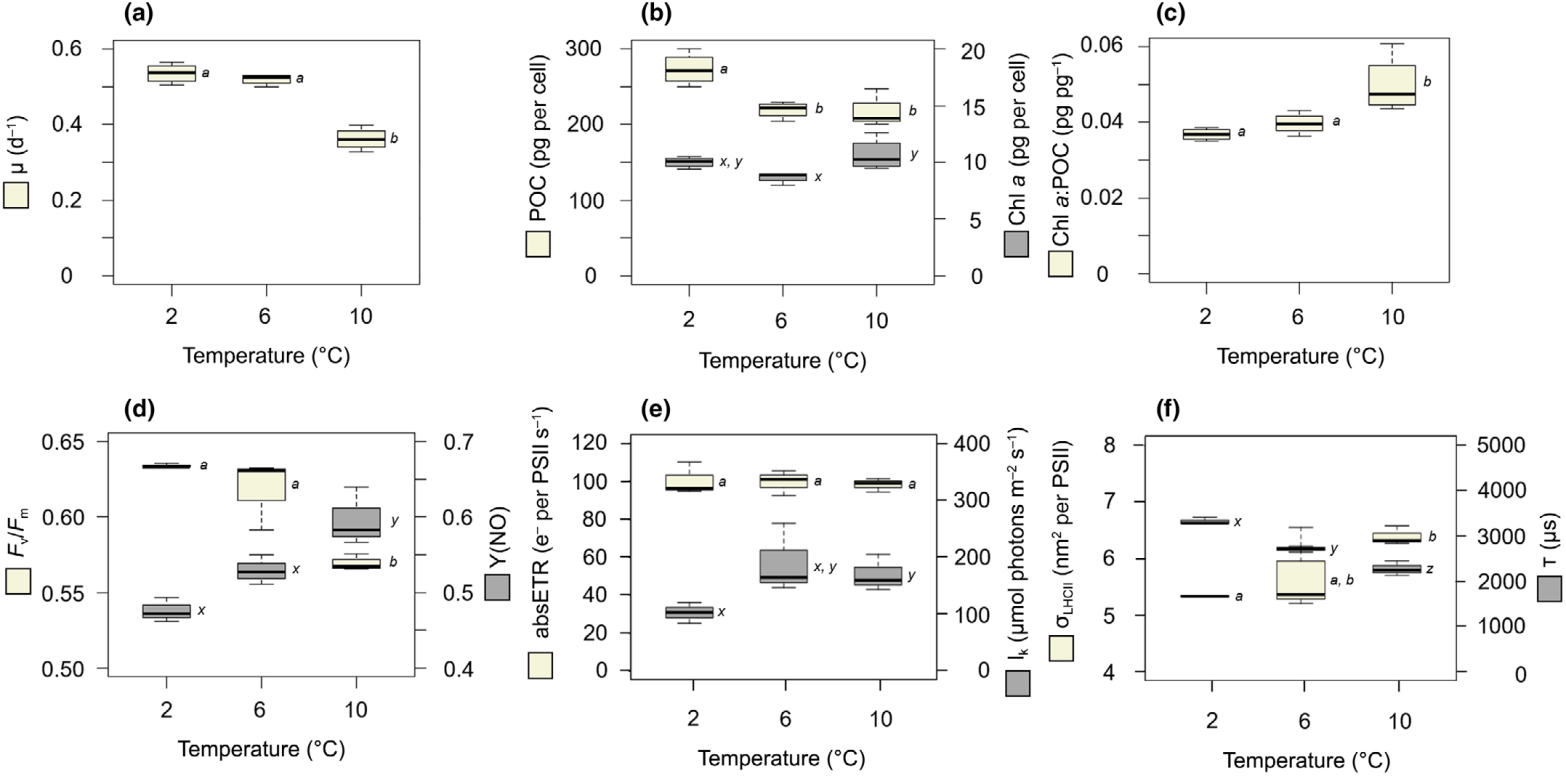
Acclimation and photophysiological parameters of *Thalassiosira hyalina* at 2°C, 6°C and 10°C. (a) Growth rate (d^−1^; beige), (b) particulate organic carbon (POC) quota (pg per cell; beige) and Chl *a* quota (pg per cell; gray), (c) Chl *a* : POC ratio (pg pg^−1^; beige), (d) photosystem II (PSII) quantum yield (*F*_v_/*F*_m_; beige) and the yield of nonregulated energy dissipation of PSII under experimental light intensity (Y(NO); gray), (e) absolute electron transport rates under experimental light intensity (absETR; e^−^ per PSII s^−1^; beige) and light acclimation indices (*I*_k_; μmol photons m^−2^ s^−1^; gray) and (f) optical absorption cross sections of PSII (σ_LHCII_; nm^2^ per PSII; beige) and re‐opening times of PSII (τ; μs; gray). The lower and upper half of boxplots represent the 25^th^ and 75^th^ percentiles, respectively. The whisker means min and max, the solid lines across each box present the median, and the colored box displays the mean. Boxplots comprise 3–4 biological replicates, and letters indicate significant differences (*P* ≤ 0.05, ANOVA).

Growth rates of *T. hyalina* were *c*. 0.52 d^−1^ at 2°C and 6°C and decreased significantly to 0.36 d^−1^ at 10°C (Fig. 1a). The POC quota was highest at 2°C (270 pg cell^−1^ and decreased significantly to 220 pg cell^−1^ at 6°C and 10°C (Fig. 1b). The Chl *a* quota was lowest at 6°C and slightly increased from 8.7 pg per cell to 10.7 pg per cell at 10°C (Fig. 1b). The Chl *a* : POC ratio was maintained at 0.038 pg pg^−1^ at 2°C and 6°C but increased significantly to 0.05 pg pg^−1^ at 10°C (Fig. 1c).

*Thalassiosira hyalina* exhibited a dark‐adapted PSII quantum yield (*F*_v_/*F*_m_) of 0.63 at 2°C and 6°C, which significantly decreased to 0.56 at 10°C (Fig. 1d). The yield of nonregulated energy dissipation of PSII (Y(NO)) under experimental light intensity increased gradually from 0.47 at 2°C to 0.60 at 10°C (Fig. 1d). The absolute electron transport rate (absETR) under experimental light intensity was maintained at 100 e^−^ per PSII s^−1^ across all temperatures, and the light acclimation index (*I*_k_) increased from *c*. 100 μmol photons m^−2^ s^−1^ at 2°C to *c*. 170 μmol photons m^−2^ s^−1^ at 10°C (Fig. 1e). The optical absorption cross section of PSII (σ_LHCII_) increased from 5.3 nm^2^ per PSII at 2°C to 6.3 nm^2^ per PSII at 10°C, and the re‐opening time of PSII (τ) decreased significantly from 3320 μs at 2°C to 2270 μs at 10°C (Fig. 1f).

### Gas flux measurements

**Fig. 2:**
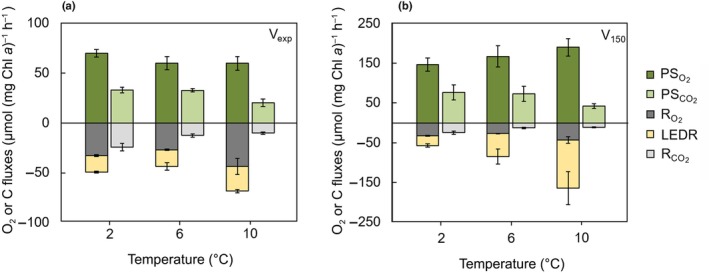
Physiological rates based on O_2_ and C fluxes normalized to Chl *a* at 2°C, 6°C and 10°C acclimation temperatures of *Thalassiosira hyalina*. Gross photosynthetic O_2_ production rates (${\mathrm{PS}}_{O_{2}}$; dark green), gross photosynthetic C‐fixation rates (${\mathrm{PS}}_{{\mathrm{CO}}_{2}}$; light green), respiratory O_2_ consumption rates in the dark ($R_{O_{2}}$; dark gray), light‐enhanced dark respiration rates (LEDR; yellow) as well as respiratory CO_2_ release rates in the dark ($R_{{\mathrm{CO}}_{2}}$, light gray) under (a) experimental light intensity of 30 μmol photons m^−2^ s^−1^ (V_exp_) and (b) 150 μmol photons m^−2^ s^−1^ (V_150_). For illustration reasons, C fluxes are presented as negative values. Error bars denote SD of three replicates (6°C and 10°C) or two replicates (2°C).

Gross photosynthetic O_2_ production rates (${\mathrm{PS}}_{O_{2}}$) rates under experimental light intensity (${\mathrm{PS}}_{O_{2}}$ V_exp_) remained at about 60 μmol O_2_ (mg Chl *a*)^−1^ h^−1^, irrespective of the acclimation temperature (Fig. 2a). Under high‐light exposure (150 μmol photons m^−2^ s^−1^), ${\mathrm{PS}}_{O_{2}}$ V_150_ was generally > 2‐fold higher than at experimental light and increased gradually with acclimation temperatures from 145 μmol O_2_ (mg Chl *a*)^−1^ h^−1^ at 2°C to 190 μmol O_2_ (mg Chl *a*)^−1^ h^−1^ at 10°C (Fig. 2b). Under experimental light intensity, gross photosynthetic C‐fixation rates (${\mathrm{PS}}_{{\mathrm{CO}}_{2}}$) remained unchanged at about 33 μmol C (mg Chl *a*)^−1^ h^−1^ at 2°C and 6°C but decreased remarkably to 20 μmol C (mg Chl *a*)^−1^ h^−1^ at 10°C (Fig. 2a). Under high‐light exposure, ${\mathrm{PS}}_{{\mathrm{CO}}_{2}}$ was also 2‐fold higher than under experimental light intensity, but exhibited the same general pattern with similar values of 75 μmol C (mg Chl *a*)^−1^ h^−1^ at 2°C and 6°C and a drop to 42 μmol C (mg Chl *a*)^−1^ h^−1^ at 10°C (Fig. 2b). Photosynthetic quotients (PQs) were *c*. 2.0 at 2°C and 6°C, while they increased to 2.5 at 10°C under experimental light intensity (Fig. S1).

Respiratory O_2_ consumption rates were always higher in the dark (R_O2_), irrespective of acclimation temperature and light exposure, due to light‐enhanced dark respiration (LEDR). Under experimental light intensities, LEDR remained constant at 2° and 6°C (*c*. 16 μmol O_2_ (mg Chl *a*)^−1^ h^−1^), but increased to 22 μmol O_2_ (mg Chl *a*)^−1^ h^−1^ at 10°C (Fig. 2a). Under high‐light exposure, LEDR increased substantially from 25 μmol O_2_ (mg Chl *a*)^−1^ h^−1^ at 2°C up to 120 μmol O_2_ (mg Chl *a*)^−1^ h^−1^ at 10°C (Fig. 2b). $R_{O_{2}}$ remained constant at 2°C and 6°C (*c*. 30 μmol O_2_ (mg Chl *a*)^−1^ h^−1^) and increased to 43 μmol O_2_ (mg Chl *a*)^−1^ h^−1^ at 10°C (Fig. 2a,b). Respiratory CO_2_ release rates ($R_{{\mathrm{CO}}_{2}}$) in the dark were highest at 2°C (24 μmol C (mg Chl *a*)^−1^ h^−1^) and decreased remarkably to 11 μmol C (mg Chl *a*)^−1^ h^−1^ at 6°C and 10°C (Fig. 2a,b). As a consequence, RQs decreased gradually from 0.7 to 0.2 between 2°C and 10°C (Fig. S1).

## Discussion

### Indications of warming‐intensified light limitation

Overall, cellular fitness, as often approximated by growth rates, did not benefit from the here tested degrees of warming. Instead, growth rates stayed constant at about 0.5 d^−1^ under the two lower temperatures (2°C and 6°C) and declined at the highest temperature of 10°C (Fig. 1a). In a previous study on the same *T. hyalina* strain using a higher light intensity (100 μmol photons m^−2^ s^−1^) but otherwise the same conditions, a clear temperature optimum with peak growth rates of 1.4 d^−1^ was observed at 8°C (Rehder *et al*., 2024). In the current study, a thermal stimulation of cell division was likely suppressed due to the here prevalent light limitation (30 μmol photons m^−2^ s^−1^). This is confirmed by light acclimation indices being consistently higher than the experimental light intensity (*I*_k_, Fig. 1e). Interestingly, *I*_k_ increased further at higher temperatures, indicating that the limitation became even stronger with warming. This response is in agreement with studies on several temperate and polar phytoplankton species as well as natural communities (Mock & Hoch, 2005; Camoying & Trimborn, 2023; Rehder *et al*., 2024; Wolf *et al*., 2024) and indicates that warming‐intensified light limitation is a general photophysiological response in phytoplankton. Hence, especially Arctic phytoplankton will likely benefit from a combined increase in temperature and light intensity, as it is projected for the future Arctic Ocean (e.g. Constable *et al*., 2022; Rehder *et al*., 2024).

### Adjustments of light harvesting maintain homeostasis in light reactions

Photophysiological parameters suggest a lower efficiency of the PSII‐associated light harvesting apparatus under increasing temperatures (Fig. 1d–f). Specifically, the observed decrease in PSII quantum yield (*F*_v_/*F*_m_; Fig. 1d), the increase in non‐regulated energy dissipation of PSII (Y(NO); Fig. 1d) as well as the lowered relative electron transport rates (rETR; Table S1) under experimental light conditions indicate a decrease in PSII performance. Despite of this, gas flux measurements indicate unchanged gross ${\mathrm{PS}}_{O_{2}}$ under all acclimation temperatures (Fig. 2a), which has also been observed in previous studies (e.g. Mock & Hoch, 2005; Rehder *et al*., 2023). To facilitate this, cells increase their pigmentation per biomass (Chl *a* : POC; Fig. 1c) and specifically enlarge the antenna complexes of PSII, indicated by the increased optical absorption cross section (σ_LHCII_; Fig. 1f). As a consequence, absETRs per PSII (Fig. 1e) were indeed maintained on a constant level, irrespective of the acclimation temperature. With this mechanism, cells increase light harvesting to compensate for a less efficient PSII, thereby maintaining gross ${\mathrm{PS}}_{O_{2}}$ on a quasi‐constant level.

PSII reoxidation times decreased under higher temperatures (τ; Fig. 1f), indicating a faster dissipation of reductant into downstream sinks. However, cells were unable to fully exploit this higher efficiency in electron transport because, under the here applied low‐light conditions, the bottleneck of photosynthesis was not reductant throughput in the first place, but rather the limiting light. This emphasizes that the capacity to adjust the light harvesting under changing acclimation temperatures is a central regulation strategy to maintain physiological homeostasis in photosynthetic light reactions, especially given the warming‐intensified light limitation.

### Light‐enhanced dark respiration increases with warming

The constant rates of ${\mathrm{PS}}_{O_{2}}$ under all tested temperatures (Fig. 2a) suggest that the production of NADPH and establishment of the PMF during the light reactions remains largely unchanged. This would also suggest that the Calvin cycle activity and thus gross photosynthetic C‐fixation (${\mathrm{PS}}_{{\mathrm{CO}}_{2}}$) likewise remains unaffected by the here applied temperatures. Indeed, up to a moderate warming (6°C), cells were able to maintain the physiological rates of ${\mathrm{PS}}_{O_{2}}$, ${\mathrm{PS}}_{{\mathrm{CO}}_{2}}$ as well as $R_{O_{2}}$ (both in light and darkness; LEDR and $R_{O_{2}}$) at process‐specific ‘comfort rates’. This is in agreement with a previous study on the temperate diatom *Phaeodactylum tricornutum* (Rehder *et al*., 2023) and indicates that also the Arctic diatom *T. hyalina* follows the same strategy to keep physiological homeostasis under moderate warming scenarios.

Since the linear electron flow of the photosynthetic light reactions typically provides a relative excess of NADPH over ATP with regard to the stochiometric requirements of the Calvin cycle (Allen *et al*., 2005; Curien *et al*., 2016; Lepetit *et al*., 2022), cells require AEFs to modulate the plastidial ATP : NADPH ratio. Our gas flux measurements indeed indicate the involvement of at least one O_2_ consuming AEF to dissipate NADPH, as signified by a 30% higher O_2_ consumption rate in the light compared to the dark (Fig. 2a), which is in line with previous observations (Falkowski *et al*., 1985; Weger *et al*., 1989; Beardall *et al*., 1994; Lepetit *et al*., 2022).

Such a light‐driven O_2_ consumption can originate from different reductant consuming pathways associated with the photosynthetic electron transport, such as PTOX (Kuntz, 2004; Peltier *et al*., 2010; Houille‐Vernes *et al*., 2011), the Mehler reaction (Mehler, 1951; Asada, 2000; Curien *et al*., 2016) as well as a re‐routing of reductant from the chloroplasts to the mitochondria (Bailleul *et al*., 2015; Lepetit *et al*., 2022; Peltier *et al*., 2024). The existence of such a metabolic coupling is well established in diatoms, green algae and land plants (Cardol *et al*., 2003; Raghavendra & Padmasree, 2003; Noctor *et al*., 2004; Rehder *et al*., 2023). In this mechanism, excess reductant is exported from chloroplasts in the form of, for example, malate and transported to the cytoplasm and the mitochondria (Heldt, 1976; Strotmann & Murakami, 1976; Kinoshita *et al*., 2011). After the reductant has been transferred to compatible carriers, specifically NADH in the mitochondria, it partly fuels the respiratory ETC, where it is ultimately transferred to O_2_ at complex IV or the alternative oxidase (Raven & Beardall, 2003). The import of reductant causes a shift in the mitochondrial redox state, and as a consequence, the citric acid cycle that normally provides NADH during catabolic operation, is suppressed (Igamberdiev, 2020). Such regulation under elevated temperature has previously been suggested for the temperate diatom *Phaeodactylum tricornutum* (Rehder *et al*., 2023) and is here also solidified by the RQ decreasing from 0.7 to 0.2 with warming (Fig. S1) as well as the high LEDR. Typically, mitochondria are thought to operate close to an RQ of 1, when respiring carbohydrates in a purely heterotrophic mode, in order to sufficiently provide reductant for respiratory ATP production (Kratz & Myers, 1955). The extraordinarily low RQ observed in *T. hyalina* can only be achieved if NADH is utilized that is not originating from the citric acid cycle, making a very strong case for the metabolic coupling being the prime compensatory mechanism to dissipate reductant, especially under warming.

Under warming, increased efficiency of microalgal carbon concentrating mechanisms has been observed (CCMs; Li & Young, 2023). A more efficient import of HCO_3_^−^ into the thylakoid lumen under these conditions consumes more H^+^ generated during the photosynthetic light reaction, affecting the PMF (Rokitta *et al*., 2022). Even if cells manage to maintain constant gross ${\mathrm{PS}}_{O_{2}}$ and PMF generation (as mentioned in the previous section), a thermally enhanced CCM activity must result in a lower ATP : NADPH ratio, and thus a higher excess of NADPH. Therefore, it can be assumed that under warming even more NADPH has to be rerouted to the respiratory ETC, explaining the even stronger downregulation of the citric acid cycle under 6°C and 10°C, as signified by the lowered $R_{{\mathrm{CO}}_{2}}$ and RQs (Figs 2a, S1). These findings are strong evidence that reductant shuttling and redox‐mediated control of mitochondrial activity are key elements in the physiological responses of microalgae to changing temperatures.

### Imbalance of ${\mathrm{PS}}_{O_{2}}$ and ${\mathrm{PS}}_{{\mathrm{CO}}_{2}}$ under strong warming

While the ratio of ${\mathrm{PS}}_{O_{2}}$ to ${\mathrm{PS}}_{{\mathrm{CO}}_{2}}$ remained stable under moderate warming, reflected by a stable PQ of *c*. 2.0 (Fig. S1), under strong warming (at 10°C), *T. hyalina* experienced a lowered ${\mathrm{PS}}_{{\mathrm{CO}}_{2}}$ despite maintained ${\mathrm{PS}}_{O_{2}}$, resulting in an imbalance of photosynthetic processes, reflected by a PQ of 2.5 (Fig. S1). In addition, the decrease of cell division and photosynthetic efficiency consistently indicates thermal stress at 10°C, which is in line with Rehder *et al*. (2024). The decrease of ${\mathrm{PS}}_{{\mathrm{CO}}_{2}}$ may originate either from detrimental temperature effects on enzymes of the Calvin cycle (Hobbs *et al*., 2013) or from further increased consumption of the PMF by a more active CCM under these conditions, as observed by Li & Young (2023). Whether these temperature responses originate from altered enzyme kinetics or increased PMF consumption should be tested in future studies. Independent of the reason for the ceasing ${\mathrm{PS}}_{{\mathrm{CO}}_{2}}$, it is in line with the general notion that processes involved in the photosynthetic light reactions are less temperature sensitive than those in the Calvin cycle (Raven & Geider, 1988) and has previously been corroborated for different microalgae in response to strong warming (Baker *et al*., 2016; Rehder *et al*., 2024). Interestingly, both phenomena, a thermal inhibition of the Calvin cycle and also stronger PMF consumption by the CCM intensify to the ‘overreduction’ of the photosynthetic ETC under warming, which was also reflected in increased Y(NO) and high LEDR (Figs 1d, 2a). During the high‐light exposure, this stimulation of LEDR was much more pronounced, likely due to a massive overreduction of the chloroplast (Fig. 2b). This emphasizes that the metabolic coupling of chloroplasts and mitochondria not only supports energy‐partitioning under heat stress, but that it is also an important regulatory strategy under high‐light stress.

## Conclusion

This study identified physiological bottlenecks under increasing temperatures, and the compensatory measures taken by the Arctic diatom *T. hyalina*. Cells increased light harvesting to compensate for the lowered photosynthetic efficiency of PSII and the warming‐intensified light limitation under elevated temperatures. Thereby, they managed to maintain absETRs, O_2_ production and thus likely PMF, irrespective of the temperature treatment. The increased LEDR under warming indicates that cells increased the photosynthetic ATP : NADPH ratio by exporting plastidial reductant into the mitochondria. This appears to fuel mitochondrial ATP synthesis directly from the chloroplast and, importantly, explains the suppressed citric acid cycle activity, that is the lowered respiratory CO_2_ loss. As a consequence, the net C retention can be maximized over a wide range of experienced temperatures, despite impaired gross C‐fixation. The outlined differential responses of O_2_ and CO_2_ fluxes can thus result in strongly diverging and unintuitive temperature response patterns. This underlines the importance to assess O_2_ and CO_2_ fluxes independently. To further investigate the here suggested metabolic coupling and its involved transport mechanisms, future studies should involve measurements of redox state kinetics as well as proteomic and transcriptomic analyses. Furthermore, field experiments should verify whether this metabolic coupling also manifests in natural phytoplankton communities. Overall, the here described mechanisms allow for the surprisingly high plasticity of Arctic phytoplankton to deal with higher temperatures and explain the stimulation of biomass accumulation under moderate warming.

## Competing interests

None declared.

## Author contributions

All authors designed the study, LR performed the experiments, LR analyzed the data, all authors discussed data interpretation and LR wrote the manuscript with input of BR, SK and SR.

## Supporting information


**Fig. S1** Photosynthetic and respiratory quotients at *in situ* light intensity of 30 μmol photons m^−2^ s^−1^ at different acclimation temperatures (2°C, 6°C and 10°C) in *Thalassiosira hyalina*.
**Table S1** Photophysiological parameters obtained from variable‐Chl *a* fluorescence measurements at different acclimation temperatures (2°C, 6°C and 10°C) in *Thalassiosira hyalina*.
Please note: Wiley is not responsible for the content or functionality of any Supporting Information supplied by the authors. Any queries (other than missing material) should be directed to the *New Phytologist* Central Office.

## Data Availability

The data that support the findings are published in the Pangaea data depository: 10.1594/PANGAEA.979803.
