# Supplementary material for: Increased light‐enhanced dark respiration under warming suggests intensified metabolic coupling in an Arctic diatom
Source: New Phytol. 2025 Jul 4;247(6):2499–506. doi: 10.1111/nph.70359 (PMC12371186; doi:10.1111/nph.70359)
Supplement: Supplementary file 1 — Fig. S1 Photosynthetic and respiratory quotients at in situ light intensity of 30 μmol photons m−2 s−1 at different acclimation temperatures (2°C, 6°C and 10°C) in Thalassiosira hyalina. Table S1 Photophysiological parameters obtained from variable‐Chl a fluorescence measurements at different acclimation temperatures (2°C, 6°C and 10°C) in Thalassiosira hyalina. Please note: Wiley is not responsible for the content or functionality of any Supporting Information supplied by the authors. Any queries (other than missing material) should be directed to the New Phytologist Central Office. [file NPH-247-2499-s001.docx]

**Supporting Information**

Article title: Increased light-enhanced dark respiration under warming suggests intensified metabolic coupling in an Artic diatom

Authors: Linda Rehder, Björn Rost, Sven A. Kranz and Sebastian D. Rokitta

Article acceptance date: 12 June 2025

The following Supporting Information is available for this article:

**Table S1.** Photophysiological parameters obtained from variable-chlorophyll *a* fluorescence measurements at different acclimation temperatures (2°C, 6°C and 10°C) in *Thalassiosira hyalina*.

**Fig S1.** Photosynthetic (PQ) and respiratory (RQ) quotients at in-situ light intensity of 30 µmol photons m^-2^ s^-1^ at different acclimation temperatures (2°C, 6°C and 10°C) in *Thalassiosira hyalina*.

**Table S1.** Photophysiological parameters at experimental light intensity of Thalassiosira hyalina acclimated to 2°C, 6°C and 10°C, obtained by fast repetition rate fluorometry. Data is presented as mean of three biological replicates ± standard deviation.

|  | **2°C** | **6°C** | **10°C** |
| --- | --- | --- | --- |
| rETR | 17.94±0.35 | 16.80±0.60 | 15.24±0.40 |
| ΦPSII | 0.56±0.01 | 0.54±0.02 | 0.50±0.01 |
| ΦNPQ | 0.02±0.00 | -0.02±0.03 | -0.04±0.02 |
| ΦNO | 0.48±0.01 | 0.53±0.02 | 0.60±0.03 |
| σ_PSII; dark_ (nm^2^ PSII^-1^) | 3.27±0.16 | 3.51±0.26 | 3.63±0.07 |

rETR, relative electron transport rate; ΦPSII, photosystem II efficiency; ΦNPQ, quantum yield of regulated energy dissipation processes; ΦNO, quantum yield of non-regulated energy dissipation processes; σ_PSII; dark_, functional absorption cross section of photosystem II in the dark in nm^2^ PSII^-1^; PSII, photosystem II.


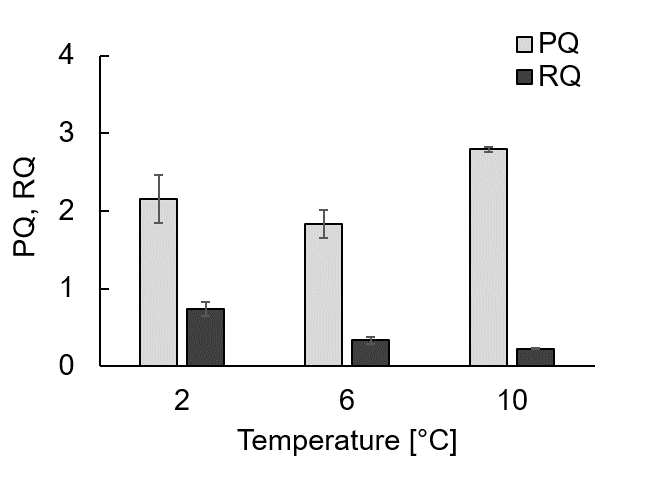


**Fig S1.** Photosynthetic (PQ) and respiratory (RQ) quotients at in-situ light intensity of 30 µmol photons m^-2^ s^-1^ at different acclimation temperatures (2°C, 6°C and 10°C) in *Thalassiosira hyalina*. Error bars denote standard deviation of 2 (2°C) or 3 (6°C and 10°C) replicates.
